# The Effect of Oral Care Product Ingredients on Oral Pathogenic Bacteria Transcriptomics Through RNA-Seq

**DOI:** 10.3390/microorganisms12122668

**Published:** 2024-12-23

**Authors:** Ping Hu, Sancai Xie, Baochen Shi, Cheryl S. Tansky, Benjamin Circello, Paul A. Sagel, Eva Schneiderman, Aaron R. Biesbrock

**Affiliations:** 1 Discovery & Innovation Platforms, The Procter & Gamble Company, Cincinnati, OH 45202, USA; hu.p@pg.com (P.H.); xie.s@pg.com (S.X.); shi.b.4@pg.com (B.S.); circello.bt@pg.com (B.C.); 2 Baby Care Clinical Group, The Procter & Gamble Company, Cincinnati, OH 45202, USA; tansky.cs@pg.com; 3 Oral Care Product Development, The Procter & Gamble Company, Cincinnati, OH 45202, USA; sagel.pa@pg.com (P.A.S.); schneiderman.e@pg.com (E.S.)

**Keywords:** oral care, transcriptomics, pathogen, bacteria, RNA-Seq, stannous fluoride, cetylpyridinium chloride, virulence factors

## Abstract

Various ingredients are utilized to inhibit the growth of harmful bacteria associated with cavities, gum disease, and bad breath. However, the precise mechanisms by which these ingredients affect the oral microbiome have not been fully understood at the molecular level. To elucidate the molecular mechanisms, a high-throughput bacterial transcriptomics study was conducted, and the gene expression profiles of six common oral bacteria, including two Gram-positive bacteria (*Actinomyces viscosus*, *Streptococcus mutans*) and four Gram-negative bacteria (*Porphyromonas gingivalis*, *Tannerella forsythia*, *Fusobacterium nucleatum*, and *Prevotella pallens*), were analyzed. The bacteria were exposed to nine common ingredients in toothpaste and mouthwash at different concentrations (stannous fluoride, stannous chloride, arginine bicarbonate, cetylpyridinium chloride, sodium monofluorophosphate, sodium fluoride, potassium nitrate, zinc phosphate, and hydrogen peroxide). Across 78 ingredient–microorganism pairs with 360 treatment–control combinations, significant and reproducible ingredient-based transcriptional response profiles were observed, providing valuable insights into the effects of these ingredients on the oral microbiome at the molecular level. This research shows that oral care product ingredients applied at biologically relevant concentrations manifest differential effects on the transcriptomics of bacterial genes in a variety of oral periodontal pathogenic bacteria. Stannous fluoride, stannous chloride, and cetylpyridinium chloride showed the most robust efficacy in inhibiting the growth or gene expression of various bacteria and pathogenic pathways. Combining multiple ingredients targeting different mechanisms might be more efficient than single ingredients in complex oral microbiomes.

## 1. Introduction

Periodontal disease is defined as a dysbiotic bacterial infection inducing a chronic inflammatory lesion of the gingiva and underlying periodontium that includes (1) the initial reversible lesion of gingivitis and (2) the advanced lesion of periodontitis where irreversible connective tissue destruction occurs. Estimates of the prevalence of gingivitis in adults range from approximately 50–100% for dentate subjects [1]. While gingivitis is a reversible condition, it is clinically relevant in that if left untreated, it can progress to the more severe form of periodontitis. Estimates of the global prevalence of periodontal disease have reported that 5–20% of the population suffers from severe periodontitis, while mild to moderate periodontitis affects the majority of adults [2,3,4]. More recently, the World Health Organization has reported that the global prevalence of severe periodontal disease, as defined by at least one site with ≥6 mm pocket depth, is approximately 19% in people above the age of 15, representing more than 1 billion cases worldwide [5]. From a global burden standpoint, severe periodontal disease is estimated to be responsible for 3.5 million years lived with disability, 54 billion USD/year in lost productivity, and a major portion of the 442 billion USD/year cost for oral disease [6].

The bacterial etiology of gingivitis and periodontitis has been well documented and attributed to the emergence of a dysbiotic subgingival microbial biofilm induced by poor oral hygiene, resulting in chronic mature undisturbed biofilm [7]. Bacterial culture-based clinical studies examining bacterial abundance in experimental gingivitis demonstrated a maturing overgrowth of dental plaque characterized by a proportional increase in *Actinomyces* species [8,9]. Furthermore, *Actinomyces viscosus* (*A. viscosus*) and *Prevotella intermedia* (*P. intermedia*) have been reported to be associated with chronic gingivitis in adults [10,11]. In 2008, a gingivitis clinical study in a twin population was reported examining the floss treatment clinical gingivitis outcomes, as well as PCR microbiologic outcomes [12]. *A. viscosus* and *P. intermedia* were statistically significantly increased in the individual twins who did not floss, and these individuals also experienced 40% greater gingival bleeding [12]. In addition, *Porphyromonas gingivalis* (*P. gingivalis*), *Tannerella forsythia* (*T. forsythia*), and *Streptococcus mutans* (*S. mutans*) were also found to be overabundant in the twins who did not floss [12]. In 2016, a bacterial community analysis in an experimental gingivitis clinical study using metagenomic methods identified the emergence of *Prevotella*, *Porphrymonas*, and *Tannerella* species associated with the onset of gingivitis [13]. A separate research study reported *P. gingivalis*, *Prevotella* species, *Fusobacterium nucleatum* (*F. nucleatum*), and *T. forsythia* to be differentially overabundant in chronic periodontitis relative to health [14]. Classically, *P. gingivalis*, *T. forsythia*, and *Treponema denticola* (*T. denticola*) have been identified as being strongly associated with one another within periodontal disease sites and have been labeled “red complex” bacteria [15]. *F. nucleatum* and *P. intermedia* are also associated with periodontal disease initiation and progression, potentially as bridging bacteria that facilitate the emergence of red complex bacteria [15]. It is important to recognize that bacteria such as *P. gingivalis*, *Prevotella* species, *F. nucleatum*, and *T. forsythia* appear to play a role in both gingivitis and periodontitis.

Over 700 bacterial species have been identified in the oral cavity [16]. An estimated 415 species are in the subgingival plaque [17], with many of these bacteria implicated in the initiation and progression of periodontal diseases [18]. Synergistic microbial communities involving both Gram-positive and Gram-negative bacteria species are involved in periodontal pathogenesis, with the interplay between bacterial species influencing the phenotypic expression of virulence factors of periodontal pathogens, such as *P. gingivalis* [19]. In communities containing all three of the species *Streptococcus gordonii* (*S. gordonii*), *F. nucleatum*, and *P. gingivalis*, about 500 proteins are differentially expressed in *P. gingivalis*, indicating a profound phenotypic change in response to the other bacterial species [20]. Nowicki et al., 2018 reported that the microbial shifts accompanying the development of gingivitis include an overabundance of *Prevotella*, *Fusobacterium*, and *Actinomyces* and increased discordance of *Tannerella* that were paralleled by the up-regulation of virulence factors in *Prevotella*, *Fusobacterium*, *Actinomyces*, and *Streptococcus* [21]. Microbial phenotypic virulence mediates periodontal disease initiation and progression. *P. gingivalis* is considered a keystone pathogen because of its ability to exert a community-wide impact that drives microbiome dysbiosis, even when it is a minor constituent in the microbial community [22]. Key *P. gingivalis* virulence factors implicated in disease pathogenesis include (a) lipopolysaccharide (LPS) implicated in toll receptor-mediated inflammation and alveolar bone loss; (b) fimbriae implicated in the adhesion to host tissues and cells that facilitate invasion, as well as bacterial co-aggregation leading to biofilm formation; and (c) proteases (e.g., gingipain, etc.) implicated in biofilm formation, the destruction of epithelial barrier function via degradation of junctional adhesion molecules, and host intracellular invasion [23,24,25,26,27]. Similarly, *P. pallens*, *T. forsythia*, and *F. nucleatum* also have LPS, fimbriae and/or surface adhesins, and proteases that have been identified as virulence factors in periodontal disease etiology [28,29,30,31,32]. *A. viscosus* and *S. mutans* play important roles in biofilm development and maturation through the production of exopolysaccharides (e.g., glucans, etc.) that play an integral role in biofilm structure. *A. viscosus*, as well as *Streptococci* species have also been shown to induce inflammatory responses in host keratinocytes mediated through bacterial lipoproteins [33,34].

Numerous oral care ingredients with antiseptic or antibacterial activities have been explored in mouth rinse and dentifrice formulations for the purpose of providing anti-plaque and anti-gingivitis benefits. These include chlorhexidine (CHX), cetylpyridinium chloride (CPC), stannous fluoride (SnF_2_), zinc chloride (ZnCl_2_), stannous chloride (SnCl_2_), sodium fluoride (NaF), and hydrogen peroxide (H_2_O_2_), all of which manifest some level of antimicrobial activity [35,36,37,38]. Arginine (Arg) has been developed as an anti-cavity ingredient that functions as a prebiotic modulating the microbiome via ammonia production by arginolytic bacteria, resulting in pH buffering, and has also been shown to have antimicrobial properties [39,40]. Potassium nitrate (KNO_3_) reduces dental hypersensitivity through nerve fiber depolarization by potassium ions and has prebiotic effects via nitrate ions [41]. Of these eight oral care ingredients that have been shown to affect the oral microflora, only SnF_2_ and CPC have a substantial body of clinical trial evidence supporting an anti-gingivitis clinical benefit [42,43]. The systematic understanding of these ingredients’ effects on specific oral bacteria transcriptomics is limited. SnF_2_ was shown to down-regulate the expression of many key enzyme genes involved in the galactose pathway, as well as key genes in the phosphotransferase system involved in sugar transport as the initial step of glycolysis in *S. mutans* [44]. H_2_O_2_ was shown to alter gene expression in *P. gingivalis*, up-regulating genes involved with detoxification such as *thiol peroxidase* (*tpx*) and *superoxide dismutase* (*sod*) [45].

As bacterial dysbiosis is a major contributor to dental disease, a deeper understanding of the response of relevant microbes to different ingredients is critical for identifying differential virulence expression leading to the development of effective oral care product solutions that meet evolving consumer demands and contribute to overall well-being. A more robust understanding of the individual effect of specific ingredients on bacterial virulence factor expression may lead to the design of specific ingredient combinations that manifest broader therapeutic antibacterial coverage, reducing the pathogenicity of oral bacteria. This work aimed to fill the existing knowledge gap by systematically evaluating the changes in bacterial functions at the transcription level. While the precise mechanisms underlying the interactions of these ingredients with oral pathogens vary, ongoing research endeavors continue to unravel their specific modes of action, paving the way for the development of advanced oral care formulations tailored to combat oral diseases effectively. Despite the widespread use of pharmaceuticals, the mechanisms underlying their effects on the oral microbiota remain poorly understood. The primary hypothesis underlying this research is that different oral care product ingredients differentially impact oral bacteria virulence gene expression. In order to test this hypothesis, a comprehensive comparative transcriptomic analysis of six different oral bacteria implicated in disease (*P. gingivalis*, *P. pallens*, *T. forsythia*, *F. nucleatum*, *A. viscosus*, and *S. mutans*) treated with nine oral care ingredients (SnF_2_, NaF, SnCl_2_, CPC, H_2_O_2_, zinc phosphate (Zn), KNO_3_, Arg bicarbonate (ArgB), and sodium monofluorophosphate (MFP) was performed, to determine and compare the responses of these oral organisms to treatment at the transcriptional level.

## 2. Materials and Methods

### 2.1. Bacteria and Culture Conditions

All bacteria were obtained from ATCC (Manassas, VA, USA, Table 1) and cultured in MTGE-anaerobic enrichment broth under anaerobic conditions with a gas mixture of 80% N_2_, 10% CO_2_, and 10% H_2_ at 37 °C. The cultures were initiated by inoculating frozen stocks. Chemicals and endotoxin-free ultra-pure water were purchased from Sigma (St. Louis, MO, USA), and MTGE-anaerobic enrichment broth was obtained from Anaerobe Systems (Morgan Hill, CA, USA). Both the broth and water were acclimated under anaerobic conditions to remove residual oxygen.

*P. gingivalis* W83 was used in preliminary experiments to determine the initial inoculation number of bacteria and the concentrations of each tested material. An overnight bacterial culture was harvested and diluted to serve as the bacterial source for the experiment. Bacterial growth was monitored at 0, 10, 24, and 48 h. Ingredient dilutions used in this research were based on previous salivary fluoride bioavailability pharmacokinetics research, where the in-use concentrations of fluoride were 1:5–1:10 at the cessation of brushing and dropped to 1:20–1:100 within 15 min following brushing [51,52].

SnF_2_ severely inhibited *P. gingivalis* growth at 2- to 32-fold dilutions of the product concentration (0.454% in toothpaste). In a separate experiment, we found that SnF_2_ inhibited *P. gingivalis* growth even at 200 μM, a 144-fold dilution [53,54]. SnF_2_, SnCl_2_, NaF, H_2_O_2_, CPC, and Zn inhibited *P. gingivalis* growth at 2- and 100-fold dilutions of the product concentrations. Noticeably, the 5-fold dilutions of H_2_O_2_ and CPC reduced the cellular masses of bacteria, resulting in insufficient RNA extraction for RNA sequencing (RNA yields were too low to make transcriptome libraries in these experiments). Consequently, we decided to treat all bacteria with the same concentrations of each compound. KNO_3_, MFP, and ArgB did not inhibit bacterial growth in the first 10 h. Therefore, we decided to apply 10-fold dilutions of their product concentrations. The final concentrations used for each ingredient are listed in the following table (Table 2).

All bacteria were inoculated at an OD_600_ of 0.2 in 10 mL of MTGE-anaerobic enrichment broth within a 15 mL Falcon conical tube containing the compound treatment. A growth curve experiment was conducted to determine the optimal treatment time point. A 3 h treatment was selected to ensure that the bacteria remained viable and that sufficient RNA could be isolated. This time point also allows for focus on the early response, avoiding long-term effects that may be associated with bacterial adaptation and mutation. After a 3 h incubation under anaerobic conditions at 37 °C, the bacteria were harvested. The bacterial pellets were flash-frozen in liquid nitrogen and shipped to BGI Americas (Cambridge, MA, USA) for RNA isolation and sequencing.

### 2.2. RNA Preparation and Sequencing

The total RNA was extracted from bacteria pellets, and ribosomal RNA was removed. The remaining RNA was fragmented into short fragments, serving as cDNA synthesis templates. The cDNA fragments were purified using the QiaQuick PCR extraction kit (Qiagen, Germantown, MD, USA) and eluted with EB buffer. Next, sequencing adapters were ligated to the short fragments. The second-strand cDNA was degraded using uracil-N-glycosylase (UNG), and the resulting product was purified with the MiniElute PCR Purification Kit (Qiagen, Germantown, MD, USA) before undergoing PCR amplification. The prepared library was sequenced with an Illumina HiSeq 4000 (Illumina, Inc., San Diego, CA, USA).

### 2.3. RNA-Seq Data Analysis

After sequencing, data cleaning was performed, and low-quality reads with adaptor sequences, contamination, and low-quality base pairs were filtered out first using cutadapt [55] (v4.0) with an average quality score of less than 30 or read lengths of less than 75% of the original length. Around 40 million cleaned reads for each sample were generated for analysis. The remaining RNA reads were mapped to the tested bacterial genome using STAR [56] (v2.7.6) and RSEM [57] (v1.3.3). Subsequence bioinformatics analysis was performed after removing ribosomal RNAs, tRNAs, and non-coding repetitive sequences after genome mapping. Statistical analysis was performed using the DESeq2 [58] (v1.34.0) package in R (v4.1.3) based on the read counts of each bacteria. The functional enrichment analysis was performed using MicrobiomeProfiler [59] (v1.0.0). The Pathview [60] (v1.34.0) package was used for mapping and visualization. PCA analyses were performed to show the clustering of samples, and the Euclidian distance was calculated and normalized to [−1, 1] to rank the treatment effect. A false discovery rate (FDR) correction was applied to adjust the significance level, and differences were considered significant at *p* ≤ 0.05. All culture and sequencing experiments were performed in a single batch, and samples were randomized during RNA preparation and sequencing to minimize batch effects. We also included eight non-treated control samples for each bacterial species. To reduce noise and mitigate environmental contamination, we excluded low-abundance genes with fewer than 10 reads per sample. This strategy enhances the reliability of our findings despite the lack of batch correction.

### 2.4. Bacterial Genome Analysis and Gene Annotation

Four of the bacterial strains, including *F. nucleatum* subsp. *nucleatum* strain VPI 4351, *P. pallens* strain NCTC 13042, *S. mutans* strain NCTC 10449, and *T. forsythia* strain FDC 338, were sequenced at ATCC using Illumina short reads and Oxford Nanopore long reads with hybrid assembly to achieve the complete genomes. The complete genome of *P. gingivalis* strain W83 was sequenced in the Institute for Genomic Research (Rockville, MD, USA) and downloaded from KEGG [49,61] (Entry: T00145). The complete genome of *A. viscosus* strain MG-1 was sequenced using Pacific Biosciences long reads with the Hierarchical Genome Assembly Process (HGAP4) de novo assembly at the Procter & Gamble Company (MOgene). All genomes were annotated using Prokka (v1.14.6) [62]. The coding genes were assigned to the KO (KEGG Orthology) IDs for microbial gene and pathway analysis using BlastKOALA (v3.0) by searching KEGG reference genomes and individual sequences linked from PubMed records of KO entries [63].

## 3. Results

### 3.1. Microbial Transcriptomics Response to Oral Hygiene Product Functional Ingredients

The transcriptomic responses of six representative oral bacteria, including *P. pallens*, *P. gingivalis*, *F. nucleatum*, *T. forsythia*, *A. viscosus*, and *S. mutans*, were measured. Among these, *P. gingivalis* and *T. forsythia* are Gram-negative pathobionts associated with periodontal disease, while *S. mutans* is a Gram-positive pathogen linked to dental caries. These bacteria were exposed to nine commonly used compounds found in oral care products at one or two concentrations mirroring those used in oral hygiene products. To ensure accuracy, experiments were conducted with four replicates for each treatment and eight for the no-treatment control across 360 treatment–control combinations for each bacterial strain.

#### 3.1.1. The Impact of Different Treatments and Dosages on Microbial RNA Yield Revealed That SnF_2_, SnCl_2_, and H_2_O_2_ Exhibited the Most Significant Reduction in Bacterial RNA Synthesis

**Figure 1:**
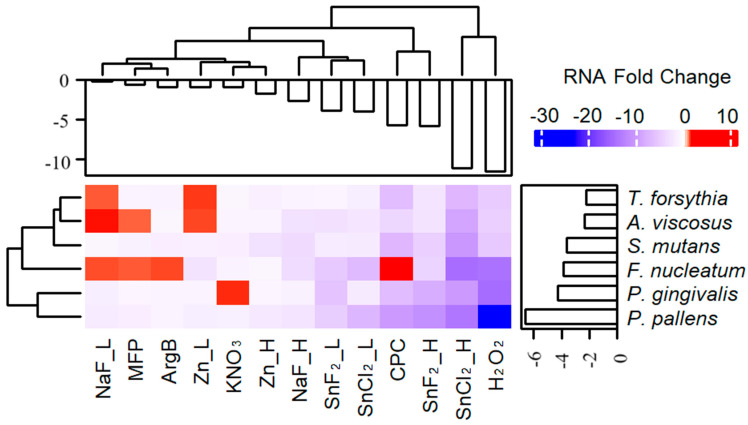
Heatmap of treatment-induced total bacterial RNA yield fold change compared to untreated control indicated that stannous and hydrogen peroxide down-regulated RNA synthesis in all tested oral bacteria.

Each treatment leg contained equivalent levels of bacteria; therefore, by measuring the total RNA yield at the end of the treatment, the treatment effect on the bacteria RNA production could be measured, which is an indication of biomass and bacterial metabolism. The fold change in the total RNA amount was calculated by comparing each treatment to the no-treatment control. Different compounds exhibit distinct effects, either inhibitory (colored blue) or stimulatory (colored red), on different bacteria (Figure 1). The top three ingredients that reduced RNA production for *A. viscosus* were SnCl_2__H, H_2_O_2_, and CPC; for *F. nucleatum*, they were SnCl_2__H, H_2_O_2_, and SnCl_2__L; for *P. gingivalis*, they were H_2_O_2_, SnCl_2__H, and SnF_2__H; for *P. pallens*, they were H_2_O_2_, SnCl_2__H, and SnF_2__H; for *S. mutans*, they were SnCl_2__H, CPC, and H_2_O_2_; and for *T. forsythia*, they were SnCl_2__H, CPC, and H_2_O_2_. SnCl_2__H and H_2_O_2_ were the best ingredients for inhibiting RNA production in all tested bacteria, while CPC reduced RNA production in all bacteria except *F. nucleatum*. Additionally, a dosage response was observed, where a higher concentration of the same compound resulted in a stronger reduction in RNA production.

#### 3.1.2. Compound and Dosage Effect Observed Based on the Differential Expressed Gene Ratio (DEGR)

**Figure 2:**
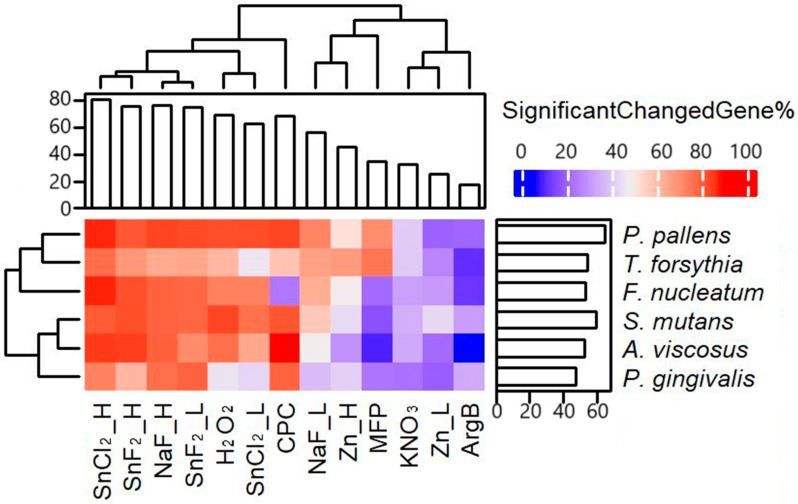
Heatmap of treatment-induced differential expressed gene ratio (DEGR) showed stannous compounds induced strong gene expression changes in all tested oral bacteria.

As the same amount of RNA was used as input for library preparation and sequencing, the gene expression change is a measurement to dissect the treatment effect independent of total RNA yield. To focus on functional genes, all the ribosomal RNA, tRNA, and repetitive non-coding regions were removed, and genes with less than ten reads per sample were further filtered out so that only robustly expressed genes were examined. A total of 12,564 genes were expressed in these six bacteria. Genes showed significant (fdr-adjusted *p*-value less than or equal to 0.05) expression changes when comparing the treatment to the no-treatment control, and then the percentage towards the total gene number was calculated and presented here as the Differential Expressed Gene Ratio (DEGR). Substantial and consistent transcriptional responses were observed across compounds and bacterial species (Figure 2). The higher expression changes are represented by the red color. Lower expression changes are represented by the blue color. Consistent with previous observations, different treatments had different effects on different pathogens. Higher compound concentrations usually induced more gene changes, with one exception being that a lower concentration of SnF_2_ induced the most gene changes in *P. gingivalis* in all treatments. The top three ingredients that led to the highest DEGR for *A. viscosus* were CPC, SnCl_2__H, and SnF_2__H; for *F. nucleatum*, they were SnCl_2__H, SnF_2__H, and NaF_H; for *P. gingivalis*, they were SnF_2__L, CPC, and NaF_H; for *P. pallens*, they were SnCl_2__H, NaF_H, and CPC; for *S. mutans*, they were H_2_O_2_, SnF_2__H, and CPC; for *T. forsythia*, they were SnCl_2__H, MFP, and SnF_2__H. Stannous compounds (SnCl_2_ and SnF_2_) led to large aggregate DEGRs across all tested pathogens. Interestingly, CPC led to strong gene expression changes in all bacteria except *F. nucleatum*.

#### 3.1.3. Ranking Treatment Effects Based on Full Gene Expression Patterns: Stannous Compounds Led to the Strongest Gene Expression Changes

**Figure 3:**
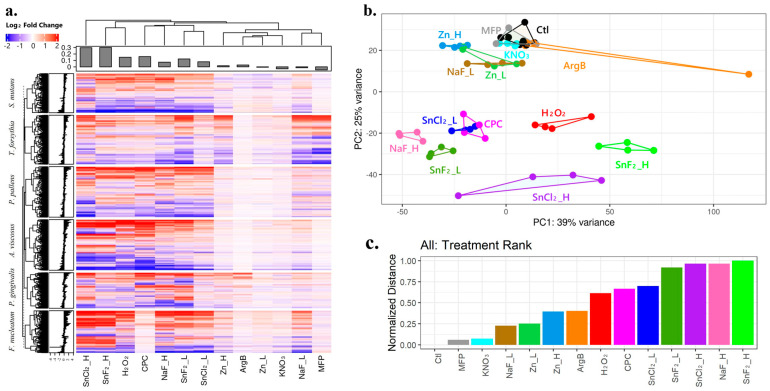
Microbial transcriptomics response to oral hygiene product ingredients is used to evaluate and rank material for treatment effect, indicating that stannous is the top treatment for these groups of tested bacteria. (**a**) Heatmap of log2 fold change of all the 12,546 genes from the six tested bacteria strains. (**b**) PCA plot of the combined gene expression data from all the 12,546 genes, showing all the tested materials and their relative distance to the control samples. (**c**) The rank treatment effect of different materials based on the normalized distance to control based on the PCA plot indicated that the stannous compounds are a top treatment for disturbing microbial gene expression, and the color matches the PCA plot.

We plotted the log2 fold change for all the 12,546 genes in the following heatmap (Figure 3a). It demonstrated a dosage effect where the higher dosage was linked with a more considerable fold change. Meanwhile, SnF_2__H and SnCl_2__H led to the strongest overall gene expression changes and clustered together with the highest average fold changes shown in the top panel. CPC had a much weaker effect on *F. nucleatum* gene expression, leading to strong gene expression changes in all the other tested bacteria species. Even though Zn tested in this study had an overall weak effect, the result demonstrated that Zn had better efficacy in targeting *T. forsythia* and *P. gingivalis* gene expression compared with the other bacteria. The PCA plot of the combined gene expression data (Figure 3b) showed that the technical duplicate for each treatment tended to cluster together showing distinctive patterns. One of the ArgB instances was much different from the other, close to the untreated control samples. The Euclidean distances of each treatment to the untreated control can be transformed within the range of 0 to 1 as an indication of similarity, or treatment effect. As shown in the bar graph (Figure 3c), SnF_2__H, NaF_H, and SnCl_2__H induced the largest gene expression changes in this experiment. We can generalize this method to evaluate the compound treatment effect by only looking at gene expression in a single species (Supplementary Figure S1a), thus evaluating the treatment effect towards each bacterium. At the same time, we can also look at each treatment and understand for which bacteria this treatment is most effective (Supplementary Figure S1b).

#### 3.1.4. Transcriptomic Responses of LPS Biosynthesis and Transportation Demonstrated That Stannous Compounds Are Potent Inhibitors

**Figure 4:**
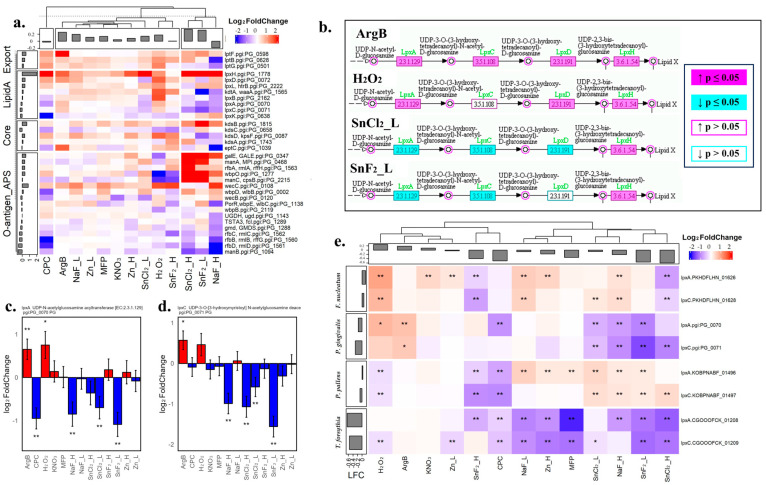
Treatment-induced transcriptomics changes in genes involved in LPS biosynthesis. (**a**) Heatmap of log2 fold change of *P. gingivalis* genes involved in LPS biosynthesis (Lipid A, Core, O-Antigen, or APS biosynthesis) and LPS export compared to no-treatment controls. (**b**) KEGG pathway mapping of the first four genes of *P. gingivalis* LPS biosynthesis pathway highlights gene expression changes induced by ArgB, H_2_O_2_, SnCl_2__L, and SnF_2__L, showing that stannous compounds down-regulated LPS biosynthesis. ArgB up-regulated LPS biosynthesis. (**c**) Bar plot of the log2 fold change of *P. gingivalis LpxA* gene, the first step for Lipid A biosynthesis, a critical component for LPS biosynthesis. (**d**) Bar plot of the log2 fold change of *P. gingivalis LpxC* gene, which is a rate-limiting gene for the LPS biosynthesis pathway. (**e**) Heat map of log2 fold change of *LpxA* and *LpxC* genes from all four tested Gram-negative bacteria. The standard error is shown as an error bar in all bar figures; a single star indicates *p*-value ≤ 0.05, and double stars indicate fdr-adjusted *p*-value ≤ 0.05.

Lipopolysaccharides (LPSs), essential components of the outer membrane of Gram-negative bacteria, are key players in inflammation and bacterial defense, particularly in *P. gingivalis*, where they act as an endotoxin and contribute to the tissue destruction and bone loss that are strongly associated with gingivitis and periodontal disease [23]. Their biosynthesis involves critical enzymatic processes, with LpxC and LpxA being the key enzymes for LPS biosynthesis [64]. We examined the gene expression profiles of *P. gingivalis* involved in the different sections of LPS biosynthesis, including Lipid A, O-antigen/APS, core biosynthesis, and export. Through heatmap analysis (Figure 4a), we observed the log2 fold change of gene expression compared to untreated bacteria. K-mean cluster analysis was used to categorize the treatment effects of the testing materials. SnCl_2__H, SnF_2__L, and NaF_H showed up in one cluster, exhibiting a strong reduction of many of these genes, especially in Lipid A biosynthesis and LPS export. In contrast, ArgB showed up-regulation in most of the genes listed. CPC belonged to a separate cluster with more genes down-regulated in O-antigen biosynthesis and not LPS transport. By mapping the genes to the KEGG LPS pathway (Figure 4b, representing the first four enzymes in the Lipid A biosynthesis pathway), we observed that SnCl_2__L and SnF_2__L down-regulated the first two critical steps. At the same time, ArgB and H_2_O_2_ up-regulated genes in this section. Figure 4c and Figure 4d display the gene expression changes for the two critical genes *LpxA* and *LpxC*, respectively. For the rate-limiting gene *LpxC*, SnF_2__L, SnCl_2__H, SnCl_2__L, and NaF_H significantly down-regulated *LpxC* expression, while ArgB significantly up-regulated it (Figure 4d). Similarly, ArgB also significantly up-regulated *LpxA* expression, with SnF_2__L being the most potent inhibitor (Figure 4c).

As LPS biosynthesis is a universal pathogenic component for Gram-negative bacteria, we further checked all of the Gram-negative bacterias’ LPS pathways (Figure 4e), with a heatmap showing the log2 gene expression of the *LpxA* and *LpxC* from all four tested Gram-negative bacteria. SnCl_2__H, SnF_2__H, and CPC are the only materials that significantly down-regulated five out of these eight genes with different potencies toward different bacteria: SnCL_2__H and SnF_2__H are the only two treatments that showed significant inhibition for the *F. nucleatum* genes; SnF_2__L, NaF_H, and SnCl_2__L are the best inhibitors for *P. gingivalis* genes; CPC and SnF_2__H work best to inhibit *P. pallens* gene expression; and many compounds can inhibit *T. forsythia* genes except ArgB and KNO_3_. Unexpectedly, the lower concentration of SnCl_2_ and SnF_2_ seems to have a better inhibitory effect on *P. gingivalis* LPS synthesis than a high concentration of the same compounds.

#### 3.1.5. Gene Expression Analysis of Key Factors Associated with *P. gingivalis* Infection: Pathogenic Protein’s Translocation and Secretion

**Figure 5:**
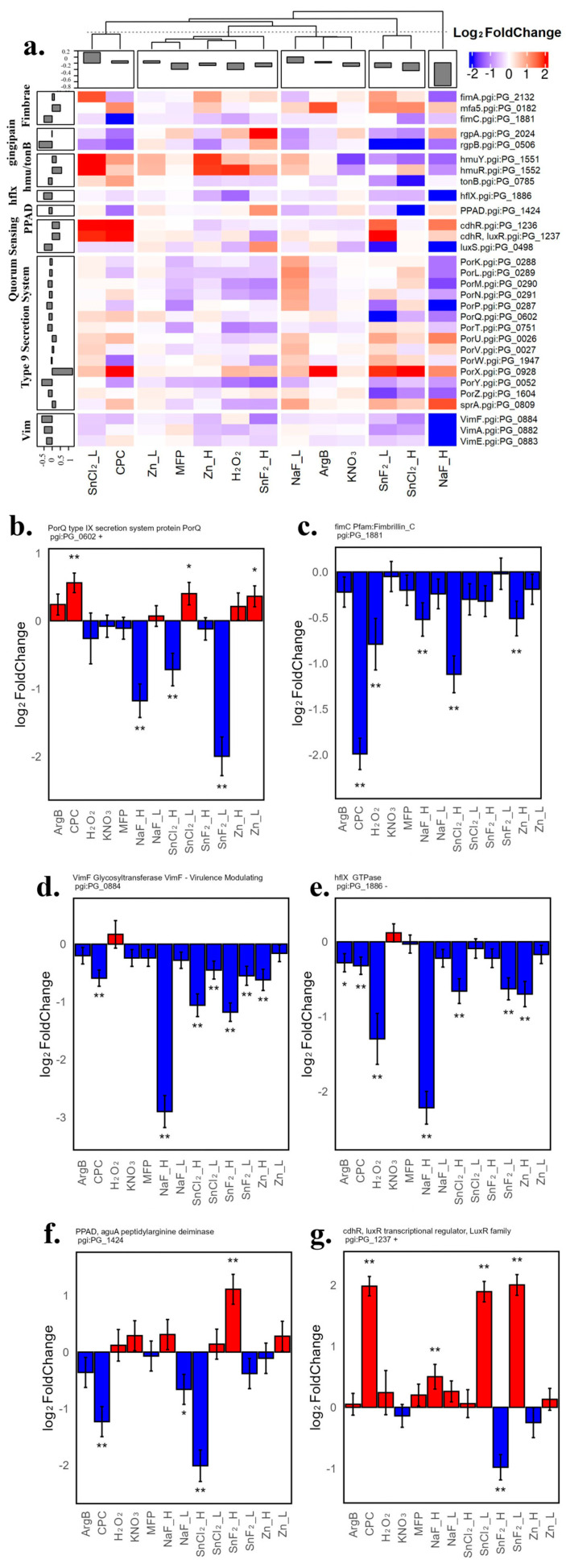
Treatment-induced transcriptomics changes in genes involved in *P. gingivalis* toxin translocation, secretion system, and infection (**a**) Heatmap of Log2 fold change of *P. gingivalis* genes involved in toxin translocation, secretion system, and infection (including Type 9 Secretion System (T9SS), *PPAD*, *gingipain*, *frimbrium*, *humY*-*tonB*, *VIM*, quorum sensing gene *LuxS*, *LuxR*, NO stress-associated gene *cdrH*, and infection-associated gene *hflX*) compared to untreated control from all tested bacteria strains. (**b**) Bar plot of the log2 fold change of *P. gingivalis* Type 9 Secretion System gene *PorQ* encoded by pgi:PG_0602. (**c**) Bar plot of the log2 fold change of *P. gingivalis fimbrium subunit C (fimC)* gene encoded by pgi:PG_1881. (**d**) Bar plot of the log2 fold change of *P. gingivalis VimF Glycosyltransferase* gene encoded by pgi:PG_0884, a key virulence modulating component. (**e**) Bar plot of the log2 fold change of *P. gingivalis hflX* gene encoded by pgi:PG_1886, a key virulence factor for infection and invasion. (**f**) Bar plot of the log2 fold change of *P. gingivalis peptidylarginine deiminase (PPAD)* gene encoded by pgi:PG_1424. (**g**) Bar plot of the log2 fold change of *P. gingivalis cdhR* gene encoded by pgi:PG_1237, also named *luxR* as a component of quorum sensing, regulating NO stress resistance. The standard error is shown as an error bar in all bar figures; a single star indicates *p*-value ≤ 0.05, and double stars indicate fdr-adjusted *p*-value ≤ 0.05.

*P. gingivalis* utilizes the Type IX secretion system (T9SS) to transport key virulence factors, including LPS, PPAD, and gingipains [65,66]. There are 14 genes identified as T9SS components expressed in this experiment. Figure 5a presents a heatmap illustrating the treatment effect on these genes, alongside fimbria formation genes, the quorum sensing gene *luxS* [67], the *LuxR* and *cdhR* genes [68], *vim* genes involved in gingipain transport and secretion [69], the *hflX* gene for bacterial infection and invasion [70], and *hmuY*-*tonB* genes enabling iron acquisition [71]. Cluster analysis revealed that NaF_H, SnCl_2__H, and SnF_2__L are the top ingredients that down-regulated many of these infection-related genes. Specifically, Figure 5b shows the gene expression of *porQ*, a key component of the T9SS apparatus, involved in the assembly and function of the secretion system. Only SnF_2__L, NaF_H, and SnCl_2__H significantly down-regulated *porQ* gene expression. Meanwhile, some virulence genes, such as *FimC* gene expression, were down-regulated by all treatments (Figure 5c), with CPC and SnCl_2__H being the top inhibitors inducing more than a 2-fold down-regulation compared to non-treated bacteria. *VimF* (Figure 5d), a gene involved in the processing and activating of gingipains, was significantly inhibited by many treatments except ArgB, H_2_O_2_, KNO_3_, MFP, NaF_L, and Zn_L. Similarly, the *hflX* gene (Figure 5e) was down-regulated by many materials except KNO_3_ and MFP. Figure 5f shows the gene expression changes of *PPAD* (*P. gingivalis* peptidylarginine deiminase), a critical virulence factor involved in the post-translational modification of proteins by converting arginine residues to citrulline [72]. Modifying the host protein structure can help *P. gingivalis* modulate various pathogenic processes, such as immune evasion and binding ability to host tissue. Three tested materials significantly down-regulated *PPAD* gene expression, in order based on the fold change from more considerable to more minor: SnCl_2__H, CPC, and NaF_L. With respect to the *CdhR* gene (Figure 5g), SnF_2__H is the only material that significantly down-regulated its expression.

#### 3.1.6. Treatment Effect of Gene Expression for Degradative Enzymes Such as Proteases, Peptidases, and Hemolysins—Stannous Compounds Showed Potent Inhibition of These Types of Virulence Factors

**Figure 6:**
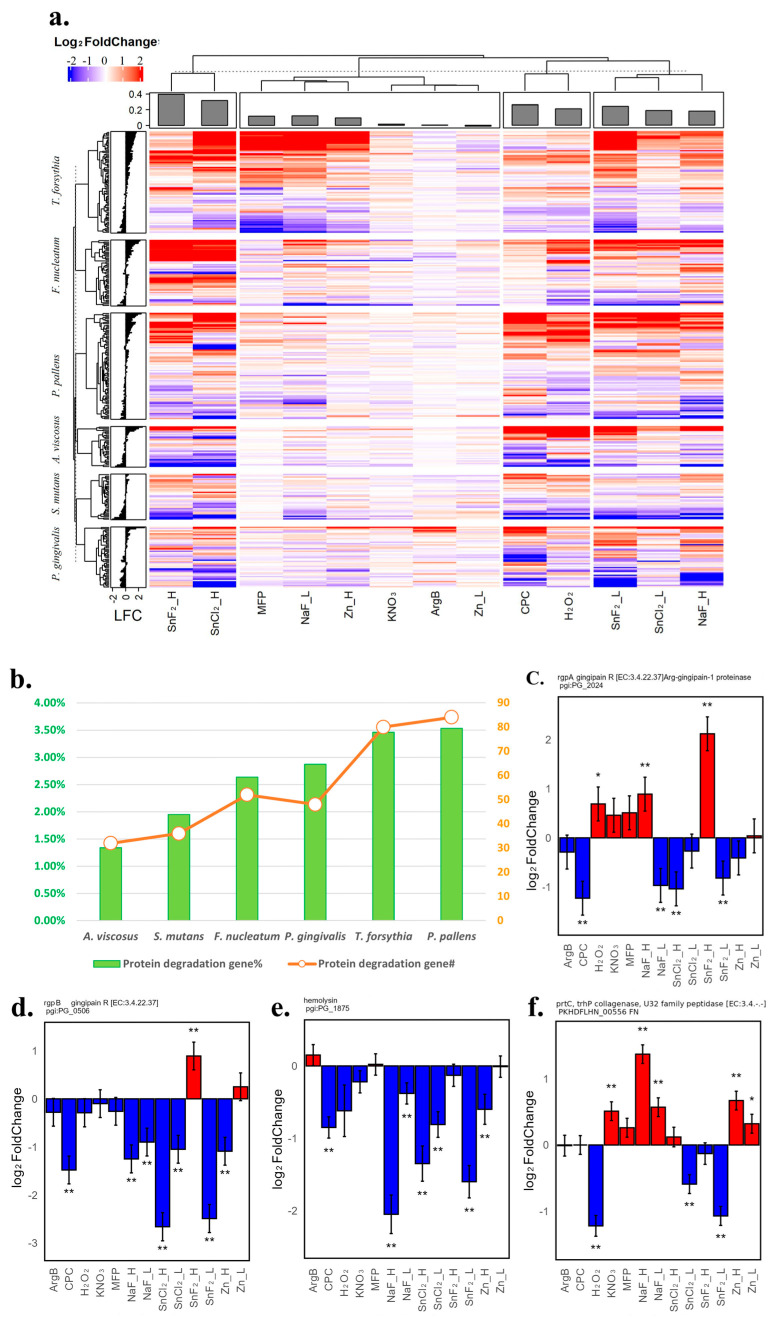
Treatment-induced transcriptomic responses of degradative enzymes including proteases, peptidases, and hemolysins. (**a**) Heatmap of log2 fold change of degradative enzymes, such as proteases, peptidases, and hemolysins, from all the tested bacteria strains compared to the no-treatment controls. (**b**) Gene number of the degradation enzymes in each bacteria genome and representational ratio towards all the genes encoded in the genome. (**c**) Bar plot of the log2 fold change of *P. gingivalis gingipain A* gene encoded by pgi:PG_2024. (**d**) Bar plot of the log2 fold change of *P. gingivalis gingipain B* gene encoded by pgi:PG_0506. (**e**) Bar plot of the log2 fold change of *P. gingivalis hemolysin* gene encoded by pgi:PG_1875. (**f**) Bar plot of the log2 fold change of *F. nucleatum* prtC *collagenase* gene encoded by PKHDFLHN_00556 [73]. The standard error is shown as an error bar in all bar figures; a single star indicates *p*-value ≤ 0.05, and double stars indicate fdr-adjusted *p*-value ≤ 0.05.

Bacterial degradation enzymes secreted by oral pathogens are crucial in the progression of oral diseases, as they break down host tissues and proteins, fostering inflammation and enabling the invasion of periodontal structures. All the genes in this category were collected through an annotation name search to create a heatmap of tissue damage degradative enzymes in Figure 6a. There was a clear dosage effect for these genes: higher concentrations of the compounds led to more transcriptomics changes. Using the K-mean algorithm, three clusters were identified with strong gene expression changes: cluster 1 contained SnF_2__H and SnCl_2__H; cluster 2 contained CPC and H_2_O_2_; and cluster 3 contained SnF_2__L, SnCl_2__L, and NaF_H. All the stannous compounds were in the strong treatment clusters with more genes down-regulated for *P. gingivalis* and *A. viscosus*, which might indicate that stannous compounds, together with CPC and H_2_O_2_, are able to better inhibit *P. gingivalis* tissue damage enzymes. Both doses of Zn, ArgB, and MFP were in the cluster with weaker transcriptomics changes.

It was also apparent that more gene copies of proteases, peptidases, and hemolysins were annotated in the tested bacteria genome of the Gram-negative bacteria than Gram-positive bacteria. Figure 6b shows the ratio of these genes in each of the six tested bacteria; the Gram-negative bacteria had more than 50 copies of the genes in this category, while the Gram-positive enzyme only had less than 40 copies. Relative to the total gene encoded in the genome, the four Gram-negative bacteria had a higher portion dedicated to encoding these enzymes than the two Gram-positive bacteria. Additionally, *P. pallens*, *T. forsythia*, and *P. gingivalis* stood out with a higher percentage of proteases, possibly highlighting their more substantial virulence. This result might indicate that the tested Gram-negative bacteria have more pathogenic potential to cause tissue damage than the tested Gram-positive bacteria. The genome adaptation helps them to thrive deep within the host tissue.

Figure 6c,d show the treatment effect for the *P. gingivalis* gingipain. CPC, SnCl_2__H, NaF_L, and SnF_2__L significantly inhibited the expression of *gingipain A* (encoded by PG_2024) (Figure 6c). The *gingipain B* gene (encoded by PG_0506) (Figure 6d) was significantly inhibited by many materials, with the top inhibition coming from SnCl_2__H and SnF_2__L with more than a four-fold reduction in its expression. For the *P. gingivalis* W83 genome, the gingipain *kgp* gene (PG_1844) has a frameshift in the coding region. Therefore, it is not included. *Hemolysin* is another important virulence factor that enables bacteria to destroy red blood cells and cause tissue damage and inflammation. Except for ArgB, MFP, KNO_3_, and H_2_O_2_, multiple tested compounds can significantly inhibit *P. gingivalis hemolysin* gene expression, with the strongest inhibition effect delivered by NaF_H, SnF_2__L, and SnCl_2__H (Figure 6e). Figure 6f shows the treatment effect for the *prtC* gene [73] in *F. nucleatum*, which encodes *collagenase*, helping bacteria to penetrate connective tissue and induce inflammation. Only H_2_O_2_, SnF_2__L, and SnCl_2__L can significantly down-regulate this gene expression.

## 4. Discussion

Many bacterial infections involve the synergistic interaction of multiple pathogenic species, which collectively enhance their ability to establish and sustain infections. A well-documented example is the oral plaque biofilm, comprising diverse bacterial species [74]. Periodontitis, a prevalent inflammatory gum disease, is commonly associated with such a polymicrobial biofilm. This biofilm typically includes *P. gingivalis*, a key pathogen implicated in tissue destruction and inflammation. Furthermore, species such as *S. gordonii* and *F. nucleatum* act as assistant pathogens or biofilm builders. *S. gordonii* facilitates the initial colonization of the biofilm, while *F. nucleatum* bridges interactions between early and late colonizers, promoting biofilm maturation and stability. These interactions not only support the structural integrity of the biofilm but also enhance the persistence and virulence of primary pathogens, thereby increasing the overall pathogenicity of the bacterial community. Treatments focusing on a single species or biological pathway can be ineffective against complex bacterial communities such as the oral microbiome.

The pathogenic mechanism of bacteria and its interplay with host tissues is a multifaceted process involving well-coordinated systems that enable the bacteria to colonize, evade host defenses, acquire nutrients, and ultimately cause tissue damage. Many of these systems are well characterized as virulence factors in the representative microbes included in this study.

**Figure 7:**
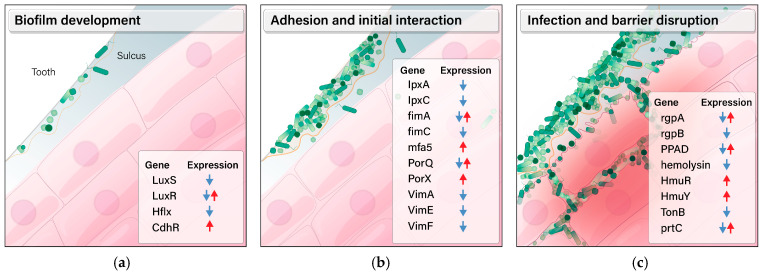
Transcriptomic changes in genes that are regulated by major oral care ingredients and involved in biofilm development, adhesion to, and infection of host cells. (**a**). Genes in biofilm development and survival. (**b**). Genes in attachment to and initial interaction with host cells, such as gingival keratinocytes. (**c**). Genes encoding products that directly degrade the cellular structure of gingiva and facilitate bacterial survival and infection. The directions of gene expression changes are based on the results observed with SnF_2_, SnCl_2_, and CPC, which had the strongest activity. Blue arrows designate down-regulation, and red arrows designate up-regulation.

In the example of *P. gingivalis*, the pathogenesis process involves several key steps (Figure 7): (1) Biofilm formation: This includes resistance development through quorum sensing (*LuxS/LuxR*, Figure 5a) that promotes bacteria aggregation [67]; the *CdhR* gene for nitric oxide stress resistance (Figure 5g) [68]; and mechanism such as Hflx (Figure 5e) that adapt bacteria to stress and treatment [70]. (2) Adhesion and initial interaction: This involves fimbriae genes (e.g., *fimA*, *fimC*, and *mfa5* genes in Figure 5a,c) that facilitate adherence to tooth surfaces and gingival tissue [75,76], as well as LPS biosynthesis pathways (e.g., *LpxA* and *LpxC* in Figure 4c,d) for binding to host receptors, including TLRs, to facilitate evasion and induce inflammation [64]. Additionally, the Type 9 Secretion System and *Vim* genes (Figure 5a,d) transport virulence factors into the extracellular environment, enhancing the bacteria’s ability to establish infection [65,66]. (3) Infection and barrier disruption: This stage includes the *PPAD* gene (Figure 5f), which modifies host proteins to enable immune evasion [72]; *gingipains* for tissue destruction [77]; and the *HmuR*, *HmuY*, and *TonB* systems (Figure 6a) that facilitate nutrient acquisition necessary for sustaining infection. *Hemolysins* disrupt the cell membrane, releasing hemoglobin for iron acquisition [71], and other proteases such as *prtC* (Figure 6f) [73] encode collagenase that helps bacteria to penetrate connective tissue and induce inflammation. Other oral pathogens, such as *T. denticola*, *T. forsythia*, and *F. nucleatum*, share many of these pathogenic mechanisms with *P. gingivalis*. These elements work together to enable *P. gingivalis* to persist in the hostile oral environment and drive chronic conditions such as periodontitis. The RNA-Seq approach in the current work allows for evaluation of the strain-specific step-by-step pathogenic mechanism from all these angles to understand the treatment, its mechanism, and limitations. SnF_2_ and SnCl_2_ showed good inhibition against these pathogenic steps. This approach can be expanded to other bacteria, as demonstrated in Figure 4c for LPS and Figure 6a for degradation enzymes.

More broadly speaking, Gram-negative bacteria in subgingival plaques continuously produce and release LPS, which binds to TLR4 receptors on cell membranes, stimulating the production of proinflammatory cytokines and oxidative stress [53,78]. LPS levels are higher in periodontitis sites compared to healthy sites in subgingival plaques, as measured using a TLR4-based method [79]. Additionally, the LPS content is elevated in gingivitis sites compared to healthy sites, as determined by both TLR4-based [80] and Limulus amebocyte lysate (LAL) methods [81]. Treatment with stannous-based toothpaste significantly reduced LPS content in subgingival plaques. Consistent with clinical observations, the expression of LPS biosynthesis genes was reduced by several tested ingredients in the current study (Figure 4), including SnF_2_, SnCl_2_, NaF, and CPC.

LPS, a key virulence determinant, can be further analyzed as it is a large and complex molecule composed of three main components: Lipid A, core oligosaccharide, and O-antigen. Lipid A, the most conserved part of LPS, anchors the molecule to the outer membrane of bacteria. The enzymes involved in Lipid A biosynthesis have been primarily characterized in *Escherichia coli* and *Salmonella* species and are well conserved across Gram-negative bacteria in different phyla. Lipid A biosynthesis begins with the linking of the precursor molecule N-acetylglucosamine to the nucleotide carrier UDP-GlcNAc (uridine diphosphate N-acetylglucosamine) to form UDP-3-O-(acyl)-GlcNAc. This process is catalyzed by the enzyme LpxA. However, the acylation product, UDP-3-O-(acyl)-GlcNAc, is not stable. LpxC stabilizes the product by deacetylating UDP-3-O-(acyl)-GlcNAc to UDP-3-O-(acyl)-GlcN. Consequently, *LpxA* and *LpxC* initiate Lipid A biosynthesis by generating the first stable intermediate [82]. As shown in Figure 4, the expression of *LpxA* and *LpxC* was inhibited by ingredients such as SnF2, SnCl_2_, and CPC. They likely reduce LPS production in Gram-negative bacteria. It is worth mentioning that LPS is a major component of the cell wall of Gram-negative bacteria, providing structural integrity and creating a permeability barrier that protects the bacterial cell from harmful molecules. A reduction in LPS biosynthesis makes bacteria more susceptible to innate immunity [83].

Proteases are essential for cellular metabolism and play a significant role in pathogenicity. They are present in all six types of oral bacteria, with Gram-negative bacteria possessing more protease genes than their Gram-positive counterparts. All four of the Gram-negative bacteria included in this study are pathogenic, relying on proteases to degrade host proteins, evade immune responses, and acquire nutrients. *P. gingivalis* is asaccharolytic and does not rely on sugars for energy. Instead, it ferments amino acids derived from proteins. *P. gingivalis* produces a variety of proteases, such as gingipains, which break down host proteins into peptides and amino acids. These smaller molecules are then utilized for energy production and growth. There are two types of gingipains: Arg-specific gingipains (*gingipain A* and *gingipain B*) and Lys-specific proteinase (*Kgp*). The oral cavity, especially the subgingival area, has an abundance of proteins from host tissues and gingival crevicular fluids. *P. gingivalis* leverages these proteins to thrive and contribute to periodontal disease. Gingipains, crucial to the bacterium’s pathogenicity, degrade cytokines and other immune signaling molecules, aiding in immune evasion. Additionally, gingipains facilitate tissue invasion and colonization by breaking down extracellular matrix components such as collagen and fibronectin.

Gingipains have been detected in various diseased tissues. They have been found in the brains of Alzheimer’s disease patients [84] and in the arteries of patients with cardiovascular diseases, where they may contribute to the formation of atherosclerotic plaques [85]. CPC, SnCl_2_, NaF, and SnF_2_ significantly inhibited the expression of *gingipain A* and *gingipain B*. Unexpectedly, high concentrations of SnF_2_ increased the expression of both *gingipains A* and *B* (Figure 6c,d). This strong up- or down-regulation suggested that these materials disturbed the metabolism of the bacteria. Regardless, up-regulation alone does not lead to increased proteolytic activity. The translation of gingipain mRNA into proteins and the processing of zymogens into active proteases require other cellular proteins. Gingipains are initially synthesized as inactive proenzymes, also known as zymogens. The activation process involves several steps. Initially, inactive gingipain precursors are synthesized within the bacterial cell. Some gene products in the type IX Secretion System (T9SS) transport and maturate the zymogen to the bacterial surface [86]. Once at the surface, specific Por secretion proteins, such as PorU, cleave the propeptide region of the gingipain zymogens [87]. This cleavage is crucial for converting the inactive zymogens into their mature, active forms. High concentrations of SnF_2_ significantly inhibited the expression of *PorU* (Figure 5a), likely impeding this maturation process and suggesting that the increased expression of *gingipains A* and *B* was impacted via a negative feedback loop.

RNA sequencing (RNA-Seq) is a powerful tool for understanding how chemical treatment affects biological functions, providing a genome-wide view of how different bacteria respond to chemical treatment rather than focusing on a single species or a few genes. Connectivity Map (CMap) [88], as a successful computational tool using gene expression profiles from cultured human cells, enables the rapid discovery of potential therapeutic material, drug repurposing, predictive toxicology, and target discovery. These findings based on RNA sequencing will help leverage this concept to create an unbiased, genome-wide map for oral pathogen interaction with ingredients. This work is the first to survey a variety of representative oral pathogens, with Gram-positive and Gram-negative strains treated with a wide range of therapeutic ingredients. It not only enables a deep mechanistic understanding through gene pathway analysis, but it also allows for screening or ranking material based on holistic impact or dissecting specific mechanisms, such as LPS, protease, or bacterial infection, offering novel insights that were unavailable with previous approaches. It also revealed the treatment specificity with respect to transcriptomic regulation, where at low dilution (1:20), CPC seems less effective against *F. nucleatum* but highly effective against all other tested bacteria. In contrast, at higher dilutions (1:3), which represent intraoral usage, CPC was found to be completely bactericidal to *F. nucleatum*, limiting the ability to observe transcriptomic changes. In support, Tada et al., 2020 previously reported that CPC at concentrations as low as 0.01% is highly bactericidal for *F. nucleatum*, killing > 70% of *F. nucleatum* cells [89]. On the contrary, MFP significantly altered gene expression in *T. forsythia* but had minimal impact on the other tested bacteria. Notably, previous research indicated that *P. gingivalis* has better oxidative stress resistance, as it is equipped with multiple mechanisms such as *VimA* and *gingipain* [69,90]. Consistently *P. gingivalis* was the least responsive to our H_2_O_2_ treatment (Supplementary Figure S1b). Building a database with RNA-Seq data from various species offers opportunities for discovery, enabling virtual screening, MOA understanding, and hypothesis generation for product or formulation development beyond oral care.

The major conclusion from this research is that oral care product ingredients (e.g., SnF_2_, NaF, MFP, SnCl_2_, CPC, H_2_O_2_, Zn, KNO_3_, and ArgB) applied at biologically relevant concentrations manifest differential effects on the transcriptomics of bacterial genes in a variety of oral periodontal pathogenic bacteria (e.g., *P. pallens*, *S. mutans*, *A. viscosus*, *P. gingivalis*, *F. nucleatum*, and *T. forsythia*). In general, the ingredients tested demonstrated dose–response efficacy, with higher concentrations resulting in more profound transcriptomic effects. SnF_2_, SnCl_2_, H_2_O_2_, and CPC were the most potent in terms of the DEGR (Differentially Expressed Gene Ratio) and ranked treatment effect of differential material based on normalized distance to controls (Figure 2 and Figure 3). This corresponds with the current understanding of clinical gingivitis benefits. Meta-analyses of randomized controlled clinical gingivitis studies examining SnF_2_ and CPC support that they are clinically proven to treat and prevent gingivitis [42,43] and have been recognized by the FDA as gingivitis actives. Across 18 randomized controlled clinical trials in 2890 subjects, 0.454% SnF_2_ dentifrice reduced gingival bleeding by 51% relative to negative control in studies with >3 months duration [42]. SnCl_2_ has been shown to manifest similar antibacterial activities to SnF_2_ [54,91]. There is a smaller body of clinical evidence that supports H_2_O_2_ as an anti-gingivitis ingredient, although there are also contradictory studies [92,93,94]. In contrast, NaF and MFP primarily function as anti-cavity ingredients, and they are frequently used as negative controls in gingivitis studies, as they do not provide meaningful anti-gingivitis benefits [42,43]. Likewise, Zn, Arg, and KNO_3_ have not been shown to provide meaningful anti-gingivitis benefits and are typically incorporated into products for plaque, tartar, breath, or sensitivity benefits. The differential effect on bacterial transcriptomics by these ingredients suggests that the specific down-regulation of bacterial virulence genes may partly explain each ingredient’s clinical ability to reduce gingival inflammation and bleeding. Accordingly, following an oral care regimen with different ingredients or with a two-step product might provide better protection for oral health, minimize resistance, and be more effective in dealing with polymicrobial problems. The RNA-Seq data can provide valuable insights to identify compounds that hit different targets or different pathogens, guiding the technology or product design. For example, a combination of SnF_2_ and H_2_O_2_ would appear to provide broader down-regulation of LPX virulence genes across multiple bacterial species than either alone (Figure 4e). This combination of ingredients has been tested in a 3-month gingivitis clinical study and found to provide superior bleeding reductions at 8 weeks and directionally better bleeding reductions at 12 weeks relative to the positive control CHX treatment [95].

Research on the effects of OTC therapeutic materials on the gene expression of specific oral bacteria is limited. Most studies utilize RT-PCR or gene chips rather than sequencing technologies such as RNA-Seq, which may yield different results than those obtained in our study. Nonetheless, we observed largely consistent patterns. For example, a study by Liu (2013) investigated the impact of CPC on gene expression in bacteria associated with halitosis [96]. They found that CPC at sub-minimum inhibitory concentration (sub-MIC) levels suppressed the expression of *mgl* (*methionine gamma-lyase*) in *P. gingivalis* and *cdl* (*L-cysteine desulfhydrase*, or *tryptophanase*) in *F. nucleatum*. In our results, CPC down-regulated both genes, with *mgl* (pgi:PG_0343) showing a 1.37-fold decrease compared to non-treated *P. gingivalis* samples (adjusted *p*-value: 0.04), while *cdl* (PKHDFLHN_00441) was down-regulated by 1.13-fold compared to non-treated *F. nucleatum* (not significant, *p*-value = 0.58). Interestingly, stannous compounds resulted in 5.56- to 11.47-fold down-regulation of *cdl* but were not able to inhibit *mgl* gene expression. These findings further strengthen our hypothesis that a combined regimen, such as toothpaste with SnF_2_ and mouthwash with CPC, may provide enhanced protection for oral hygiene, particularly against halitosis. Multiple studies have investigated the effects of stannous compounds, particularly SnF_2_, on gene expression in oral bacteria. For instance, Shi et al. (2018) examined the impact of SnF_2_ on *S. mutans* and *A. viscosus* using microarray analysis [44]. Their findings revealed that SnF_2_ significantly inhibited genes involved in the galactose pathway, the phosphotransferase system for sugar transport, and lactic acid synthesis. In our study, we also observed a significant down-regulation of these pathways by SnF_2_; specifically, the *LacABCD* genes in *S. mutans* (*LacA*: MMOMBGGD_00663, *LacB*: MMOMBGGD_00664, *LacC*: MMOMBGGD_00665, *LacD*: MMOMBGGD_00666) were down-regulated by 5.35- to 9.99-fold. Recent research has utilized metagenomics and metatranscriptomics to explore microbial gene expression, allowing for a more comprehensive understanding of microbial interactions within complex biofilm structures, as opposed to our method, which focuses on one bacterium and one ingredient at a time. For example, Gumber et al. (2022) employed metatranscriptomics to analyze gene expression in supragingival plaque samples after 14 days of using toothpaste containing MFP and SnF_2_ [97]. They found that SnF_2_ significantly down-regulated pathways associated with biofilm formation, cell adhesion, and quorum sensing, demonstrating greater efficacy than MFP.

While the RNA-seq approach offers valuable insights, it has several drawbacks. It is relatively expansive. The data are susceptible to batch effects and require sophisticated bioinformatics resources for analyzing large datasets. Additionally, the current method captures only a snapshot of gene expression at a specific time point and dosage, without revealing whether transcripts are translated into proteins or how those proteins interact in cellular processes. In this study, the focus was on a short-term, 3 h treatment to capture the expression of most bacterial genes. However, this approach does not address long-term changes associated with bacterial adaptation and genetic mutations. Moreover, since bacteria in the growth phase were used, it does not account for conditions when bacteria are dormant and exhibit limited gene expression, or the fact that some bacteria genes might not be expressed under the testing conditions. Furthermore, relying solely on transcriptomic data neglects potential protein-level or functional changes that may occur. To fully validate these findings and assess the long-term effects of these ingredients on the oral microbiome, additional in vivo studies are essential. Another potential limitation of this study is the focus on the six single species of bacteria. Future studies could include multispecies evaluations and explore the effects on bacteria with extended exposure to the oral care product ingredients as well as evaluations using metagenomic or metatranscriptomic techniques. Despite these limitations, the potential to explore diverse bacterial mechanisms offers a promising avenue for designing more targeted, effective, and long-lasting therapies for oral and other microbial diseases. 

## 5. Conclusions

Various oral care ingredients affect the gene expression of different oral pathogenic bacteria. The current data indicate that SnF_2_, SnCl_2_, and CPC have the most robust efficacy in inhibiting the growth or gene expression of various bacteria and pathogenic pathways. In contrast, ArgB, KNO_3_, and Zn have less impact. A combination of different ingredients might deliver better protection against different pathogens. Furthermore, the understanding generated from this study might guide technology identification, product design, and methods for exploring bacteria/host interactions. The RNA sequencing method provides valuable opportunities to reveal complex interactions and responses among different pathogens.

## Figures and Tables

**Table 1:** The bacterial strains tested in this study.

Bacterial Strain	ATCC
*Prevotella pallens* NCTC 13042	700821 [46]
*Streptococcus mutans* NCTC 10449	25175 [47]
*Actinomyces viscosus* MG1	43146 [48]
*Porphyromonas gingivalis* W83	BAA-308 [49]
*Fusobacterium nucleatum* subsp. *nucleatum* VPI 4351	23726
*Tannerella forsythia* FDC 338	43037 [50]

**Table 2:** The compounds and concentrations used in this study.

Compound	Concentration in Oral Care Products	High Estimated In-Use Concentration (Dilution of 1:2–1:10)	Low Estimated Post-Use Concentration (Dilution of 1:20–1:100)
Stannous Fluoride (SnF_2_)	0.454% Dentifrice	0.0908 (1:5) (SnF_2__H)	0.00908 (1:50) (SnF_2__L)
Sodium Fluoride (NaF)	0.243% Dentifrice	0.0908 (1:3) (NaF_H)	0.00908 (1:30) (NaF_L)
Sodium monofluorophosphate (MFP)	1.14% Dentifrice	0.114 (1:10)	-
Cetylpyridinium Chloride monohydrate (CPC)	0.1% Rinse	-	0.002 (1:50)
Hydrogen Peroxide (H_2_O_2_)	3.0% Dentifrice	-	0.06 (1:50)
Zinc Phosphate (Zn)	1.0% Dentifrice	0.5 (1:2) (Zn_H)	0.05 (1:20) (Zn_L)
Stannous Chloride (SnCl_2_)	0.5% Dentifrice	0.09 (1:5) (SnCl_2__H)	0.09 (1:50) (SnCl_2__L)
Potassium Nitrate (KNO_3_)	5.0% Dentifrice	0.5 (1:10)	-
Arginine Bicarbonate (ArgB)	8.0% Dentifrice	0.8 (1:10)	-

## Data Availability

Data and analyses are available upon request. The RNA-Seq samples’ information and sequences have been deposited into the NCBI SRA, the genome assembly for the *A. viscosus* strain MG-1 has been deposited into NCBI GenBank (Submission ID: SUB14934034; BioProject ID: PRJNA1199624).

## Supplementary Materials

The following supporting information can be downloaded at: https://www.mdpi.com/article/10.3390/microorganisms12122668/s1, Figure S1: Ranking Treatment Effect Based on the Normalized Distance from PCA.
