# Supplementary material for: The Effect of Oral Care Product Ingredients on Oral Pathogenic Bacteria Transcriptomics Through RNA-Seq
Source: Microorganisms. 2024 Dec 23;12(12):2668. doi: 10.3390/microorganisms12122668 (PMC11728304; doi:10.3390/microorganisms12122668)

Supplementary Material:

Figure S1. Ranking Treatment Effect Based on the Normalized Distance from PCA. (1a) Ranking Map with Normalized Euclidean distance to No-treatment Control Samples) shows the strength of the test material based on their overall gene expression changes for each bacterial or gene expression changes from all the bacteria. (1f) Ranking Map shows the ranking of the effectiveness of each treatment on different bacteria strains.

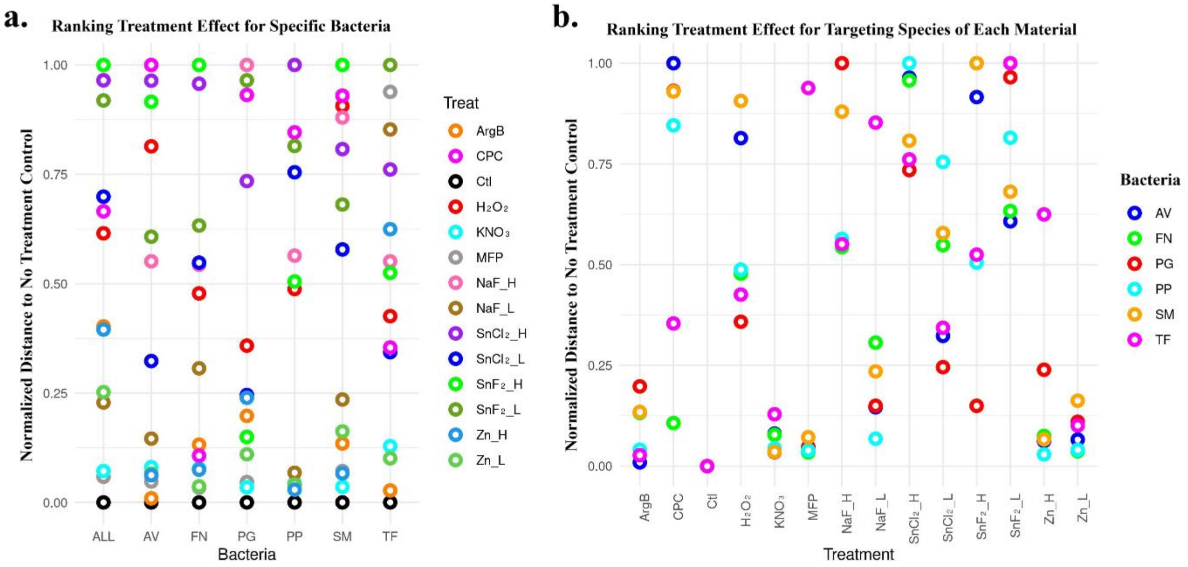

Supplement: Supplementary file 1 [file microorganisms-12-02668-s001.zip › microorganisms-3363839-supplementary.pdf]
